# Chinese herbal medicine for the treatment of cough variant asthma: a study protocol for a double-blind randomized controlled trial

**DOI:** 10.1186/s13063-018-3073-x

**Published:** 2019-01-03

**Authors:** Rong-rong Fan, Ze-huai Wen, Da-wei Wang, Rong-yuan Yang, Ai-hua Ou, Lian-shun Jin, Zhong-de Zhang, Yun-tao Liu

**Affiliations:** 1 The Second Clinical Medical College of Guangzhou University of Chinese Medicine, Guangzhou, 510405 China; 2 The Second Affiliated Hospital of Guangzhou University of Chinese Medicine, Guangzhou, 510120 China; 3 Lingnan Zhenshi Miscellaneous Diseases School, Guangdong Provincial Hospital of Chinese Medicine, Guangzhou, 510120 China; 4 Key Unit of Methodology in Clinical Research, Guangdong Provincial Hospital of Chinese Medicine, Guangzhou, 510210 China

**Keywords:** Chinese herbal medicine, Cough variant asthma, Randomized controlled trial

## Abstract

**Background:**

Cough variant asthma (CVA) is one of the leading causes of chronic coughing. The main treatment is currently anti-inflammatory medication. However, the coughing may return or be aggravated and lung function may deteriorate once the anti-inflammatory treatment is stopped. The effect of Chinese herbal medicine (CHM) on chronic coughing is remarkable, but high-quality evidence supporting its effectiveness is still lacking. This trial aims to evaluate the safety and efficacy, especially the long-term efficacy, of CHM plus anti-inflammatory medications for the treatment of CVA.

**Methods/design:**

A randomized placebo-controlled double-blind trial will be conducted. It will consist of a 3-month intervention followed by a 6-month follow-up period.

The target sample size is 60 patients with CVA who are between 18 and 70 years old. The eligible subjects will be allocated randomly into the experimental or control group in a ratio of 1:1. Patients in the experimental group will take CHM granules (4.9 g twice daily), while patients in the control group will be given a matched placebo. An administration of salmeterol/fluticasone propionate combination for 12 weeks will be the basic therapy for the two groups. The primary outcome is the cough visual analog scales (CVAS). The secondary outcomes include quality of life, rate of symptom relapse, lung function, and blood tests. A safety assessment will also be performed during the trial.

**Discussion:**

The evidence gathered by the trial will be a valuable addition to informing treatment options for patients with CVA.

**Trial registration:**

http://www.chictr.org.cn, ID: ChiCTR-IOR-16009148. Registered on 3 September 2016.

**Electronic supplementary material:**

The online version of this article (10.1186/s13063-018-3073-x) contains supplementary material, which is available to authorized users.

## Background

Cough variant asthma (CVA) is the most common cause of chronic cough [1–3]. According to a Chinese epidemiological survey, CVA accounts for 1/3 of cases of chronic cough [4]. In Japan, this proportion reaches 42.2% [5]. Long-term and constant coughing seriously affects the quality of life of patients with CVA. Such patients tend to be more depressed and anxious than classic asthma outpatients, on average [6]. Currently, maintenance therapy with a combination of inhaled corticosteroid and long-acting β2-agonist (ICS/LABA) is recommended for the treatment of CVA [7–9]. After discontinuing the anti-inflammatory treatment, cough symptom scores may increase, and lung function and eosinophilic airway inflammation may become aggravated and return to their baseline levels. The cumulative relapse rate of symptoms is as high as 67% after discontinuing treatment for 6 months [7].

Chinese herbal medicine (CHM) is widely used for CVA in China. It is reported that CHM plus ICS/LABA is better at reducing cough symptom scores, improving lung function, and improving quality of life compared with ICS/LABA alone [10–12]. Studies have also suggested that CHM plus ICS/LABA could further reduce levels of serum inflammatory factors, such as IL-10, IL-17, and IL-4/IFN-γ, compared to treatment with ICS/LABA alone [13, 14]. Furthermore, CHM plus ICS/LABA is better at reducing levels of airway neurotransmitters in the sputum than ICS/LABA alone [11]. Another study showed that compared to ICS/LABA alone, CHM plus ICS/LABA was superior at inhibiting airway remodeling in patients with CVA [15]. Although many clinical studies have proven that CHM plus ICS/LABA is superior to ICS/LABA alone for the treatment of CVA, the methodological quality of these studies was low because of the absence of sample size calculations, lack of definite random methods, unclear use of blinding methods, and so on. Furthermore, in only one study did participants undergo a follow-up period after stopping treatment [12]. How do cough symptoms, lung function, and airway inflammation change after stopping treatment with CHM plus ICS/LABA? Do these outcomes show a greater improvement after treatment with CHM plus ICS/LABA compared to after treatment with ICS/LABA alone? These questions remain to be determined.

A CHM prescription that was developed by Professor Zhang consists of ephedra (*mahuang*), asarum (*xixin*), fructus schisandrae (*wuweizi*), folium eriobotryae (*pipaye*), radix peucedani (*qianhu*), pummelo peel (*huajuhong*), folium perillae (*zisuye*), radix sileris (*fangfeng*) and radix asteris (*ziwan*). From the theory of traditional Chinese medicine, this prescription can warm the lungs, dispel wind, relieve coughing, and reduce the production of sputum. Most Chinese herbs in the prescription have been proven to be useful in the treatment of asthma. For example, it has been proven that the effective components of ephedra (*mahuang*) can decrease the secretion of IL-5, IL-13, and IL-8, alleviate the infiltration of eosinophils in the bronchoalveolar lavage fluid, inhibit airway remodeling, and inhibit the proliferation of airway smooth muscle cells [16–19]. Asarum (*xixin*) and fructus schisandrae (*wuweizi*) are often used in combination in Chinese medicine. Animal experiments have shown that the effective components of these two herbs may prolong the coughing latent period and decrease coughing frequency [20]. An extract of fructus schisandrae can also increase antioxidant levels and reduce oxidative damage. This also suggests that it may be helpful in the treatment of asthma [21]. It has been reported that folium eriobotryae (*pipaye*) has anti-inflammatory and immunoregulatory effects [22, 23]. An injection of folium eriobotryae can reduce the number of CD4+ cells, enhance the function of CD8+ cells, and regulate the CD4+/CD8+ imbalance [24]. The Th17/Treg cell balance has recently been discovered to be important in the pathogenesis of asthma. It has been reported that radix peucedani (*qianhu*) can alleviate airway inflammation and reduce airway hyperresponsiveness by regulating the imbalance of Th17/Treg cells [25]. In China, pummelo peel (*huajuhong*) is one of the most widely used Chinese herbs for respiratory diseases. Animal experiments have shown that pummelo peel can eliminate phlegm and relieve coughing, while also having anti-inflammatory, antioxidant, and immunomodulatory effects [26–29]. In addition, the other herbs in the prescription, folium perillae (*zisuye*), radix sileris (*fangfeng*), and radix asteris (*ziwan*), have anti-inflammatory effects as well. In general, the prescription may alleviate the symptoms or causes of asthma in multiple ways.

Based on a review of the literature and clinical observation, we predicted that our prescription plus ICS/LABA may be better at relieving coughing, improving lung function, and alleviating airway inflammation compared to ICS/LABA alone, even after the discontinuation of treatment. An observational study with a small sample size of 20 was carried out at a respiratory outpatient department. The control group was treated with ICS/LABA, while the experimental group was treated with ICS/LABA plus CHM. During the 12-week treatment period, the cough visual analog scales (CVAS) decreased from 5.13 ± 1.42 (mean ± standard deviation) to 1.21 ± 0.93 in the control group and from 5.11 ± 1.10 to 0.8 ± 0.69 in the experimental group. However, at the end of the 24-week follow-up phase, the CVAS increased to 4.50 ± 2.72 in the control group and 2.36 ± 2.54 in the experimental group. In addition, the cumulative percentages of patients whose symptoms worsened were 60% in the control group and 20% in the experimental group at the end of the follow-up period. We observed that ICS/LABA plus CHM was better for the treatment of CVA than ICS/LABA alone, not only during the 12-week treatment phase but also during the 24-week follow-up phase. Therefore, we have designed a randomized controlled trial to evaluate the long-term efficacy and safety of CHM plus ICS/LABA for the treatment of CVA.

## Methods/design

### Study design

**Fig. 1:**
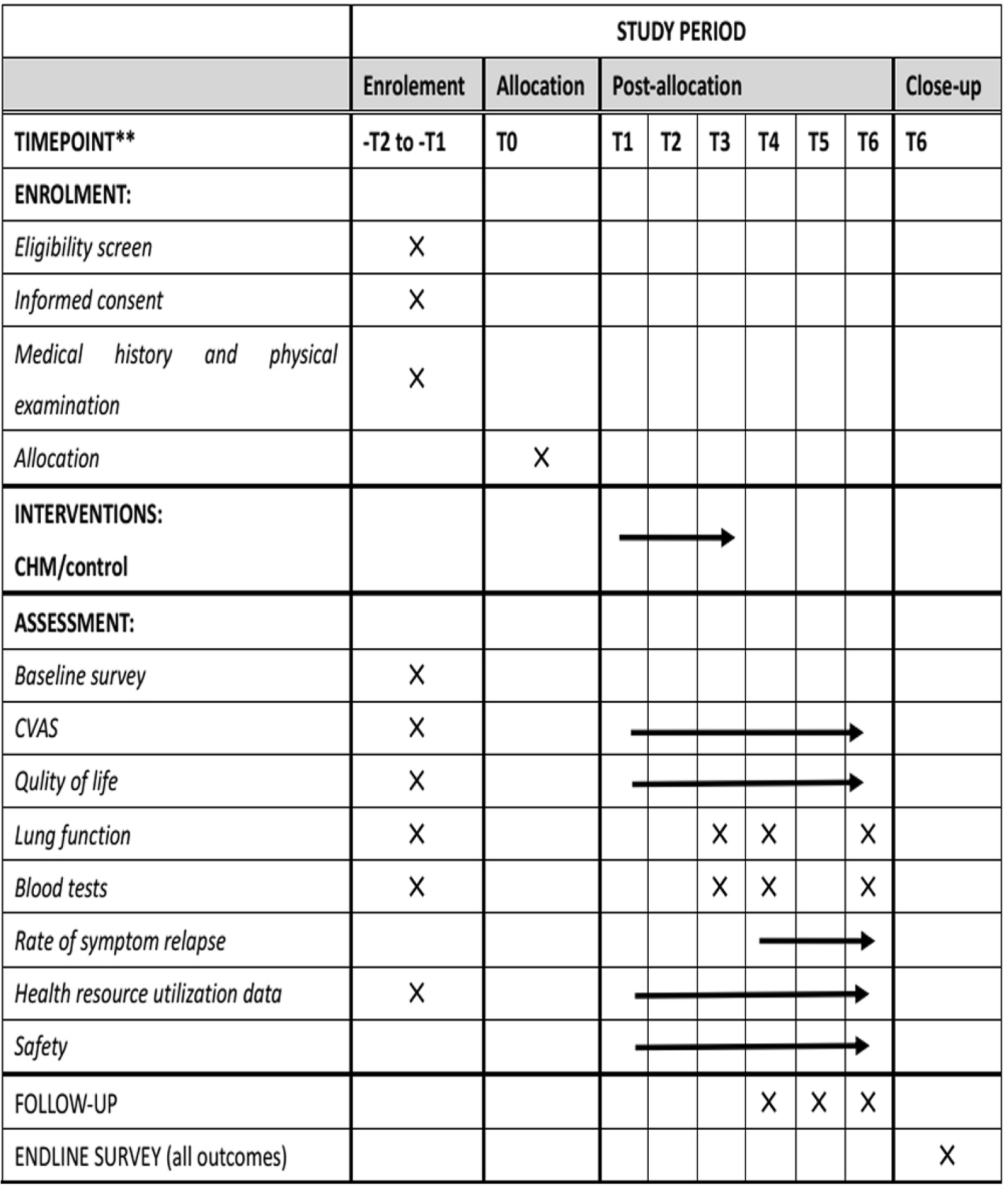
Spirit figure

This is a single-center double-blind randomized controlled trial. The trial has been approved by the Institutional Ethics Committee of Guangdong Provincial Hospital of Chinese Medicine (GPHCM; B2016–097-01) and registered in the Chinese Clinical Trial Registry (ChiCTR-IOR-16009148). The trial aims to investigate the additional benefit and safety of oral CHM compared to salmeterol/fluticasone propionate combination (SFC) alone for the treatment of CVA, with special consideration of long-term efficacy. This study protocol conforms to the Standard Protocol Items: Recommendations for Interventional Trials guidelines (Fig. 1 and Additional file 1).

### Trial procedure

**Fig. 2:**
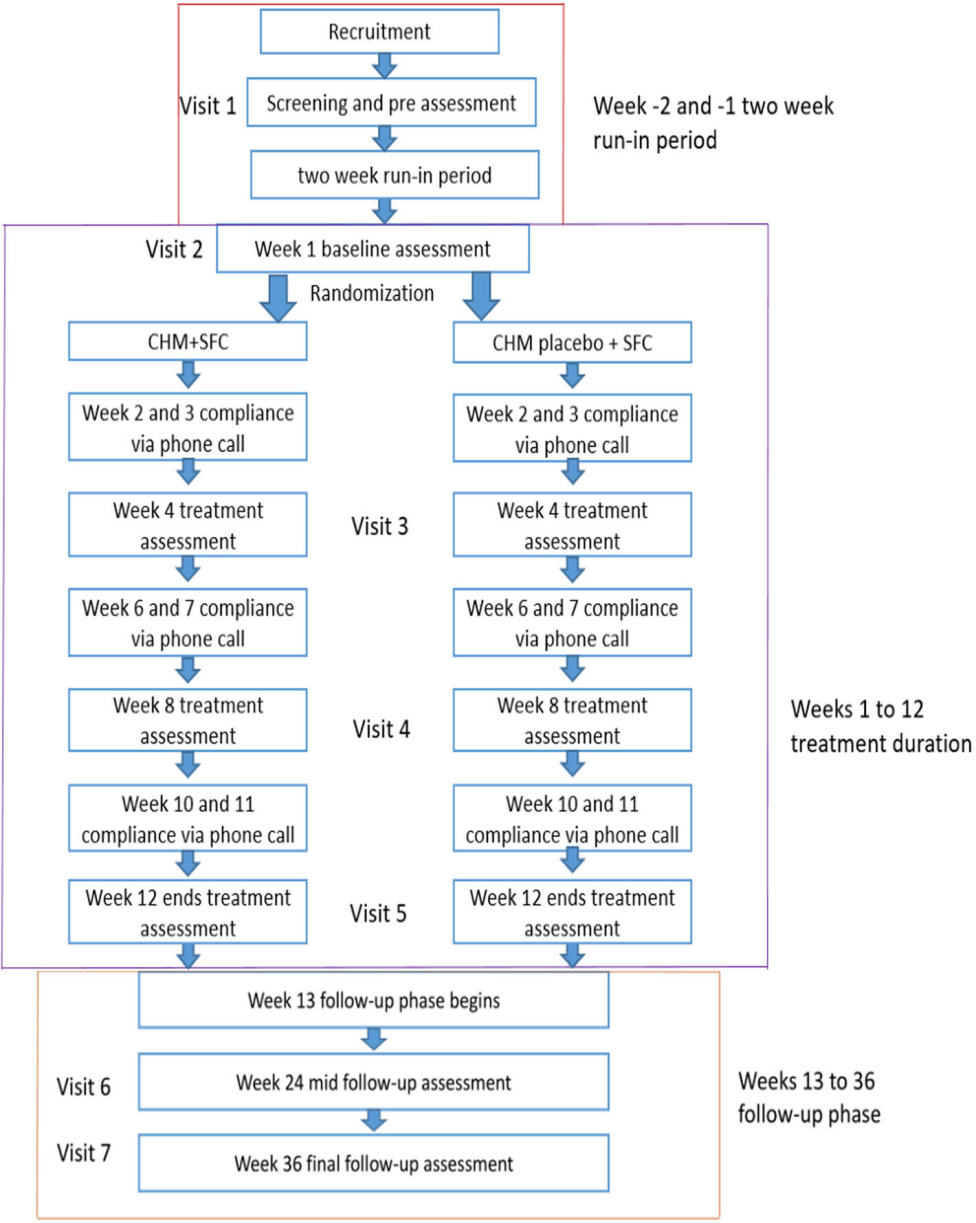
Trial flow chart. CHM Chinese herbal medicine, SFC salmeterol/fluticasone propionate combination

The entire trial consists of a screening assessment, a 2-week run-in period (weeks −2 and −1), a 12-week treatment period (weeks 1 to 12), and a 24-week follow-up period (weeks 13 to 36) (Fig. 2).

Potential participants will be invited to an initial assessment (first visit) consisting of screening for inclusion and exclusion criteria. If eligible, participants will be asked for informed consent and will undergo assessments of CVAS and quality of life, as well as a full blood count, blood tests on kidney and liver function, an electrocardiogram, and a chest radiograph examination. Then they will commence a 2-week run-in period. During this period, the participants’ use of asthma-related medication and CHM will be restricted. Participants will be provided with a salbutamol inhaler (Ventolin 5 mg/ml, GlaxoSmithKline, UK), to be used as required during this period to ease coughing until the treatment period begins. In the 2-week run-in period, the participants will be required to record the severity of their cough each day using the CVAS. After the run-in period, participants will undergo another set of baseline assessments (second visit), including assessments of quality of life, lung function, and blood tests.

After the baseline assessments, participants will be randomly allocated to receive 12 weeks of CHM plus SFC or placebo plus SFC. CVAS will be self-reported by the participants and recorded daily in a diary during the treatment period. Face-to-face assessments with blinded assessors will be scheduled at weeks 4 (third visit), 8 (fourth visit), and 12 (fifth visit). These will collect additional data, including lung function assessments and blood tests, as well as evaluations of quality of life, safety, and drug usage. Participants will be interviewed by telephone at weeks 2, 6, and 10 to determine adherence to the medication regulations and to report adverse events (AEs). The frequency of visits and phone calls can be increased if necessary.

During the 24-week follow-up phase, participants will continue to record CVAS daily in their diaries and document their utilization of health resources (including drug therapy use). They will attend follow-up assessments at weeks 24 and 36 (sixth and seventh visits). At each visit, quality of life, lung function, AEs, and drug usage will be assessed and participants will undergo blood tests.

### Participants

The participants will be recruited from the respiratory outpatient department of GPHCM. Poster, newspaper, and Internet advertisements will also be used for recruitment. Participants will be included only if they satisfy all the following inclusion criteria: (1) the diagnostic criteria for CVA [9], (2) between 18 and 70 years of age, and (3) CVAS ≥ 2.

Patients with one or more of the following conditions will be excluded: (1) systematic corticosteroid treatment, bronchodilator treatment, or CHM treatment in the last 4 months; (2) serious acute or chronic organic disease or mental disorder; (3) pregnant, breastfeeding, or planning a pregnancy; (4) known sensitivity to the intervention drugs of the trial; and (5) participating in other trials or participation in a trial within the last month.

### Interventions

#### Experimental group

The oral CHM granule will be produced by Tian Jiang Pharmaceutical Co., Ltd. (Jiangyin, Jiangsu, China). The herbs in the prescription will be mixed, cooked, filtered, and pressure spray-dried to form granules. The granules will be packaged into small single-dose sachets, each weighing 4.9 g. Participants in the experimental group will take two sachets twice a day for 12 weeks, dissolving each dose in warm boiled water. At the same time, patients will take SFC (50/250 μg, GlaxoSmithKline, England) twice a day for 12 weeks.

#### Control group

Placebo granules will be produced and packaged by the same manufacturer. They will consist of starch with no active ingredients. The placebo will be matched as closely as possible in appearance and taste to the real granules by adding various edible pigments. The dosage and administration of the placebo and SFC will be the same as in the experimental group.

### Randomization, allocation concealment, and blinding

Eligible patients will be randomly allocated into the experimental group or the control group in a ratio of 1:1 through the central web-based interactive randomization service system run by GPHCM. A block randomization procedure will be performed by the Key Unit of Methodology in Clinical Research at GPHCM using SAS 9.2 software (SAS Institute Inc., Cary, USA).

The allocation will be concealed until the trial has closed and the analysis has completed. The patients, investigators, outcome assessors, and statisticians will not know the intervention allocation. The randomization schedule and blinding codes will be kept strictly confidential until the statistical analysis has completed. Blinding will be ensured by using a matched placebo granule identical in color, shape, and taste to the intervention granule. The quality of the CHM granule and the matched placebo will be rigorously controlled according to Chinese good manufacturing practices, and will be tested and verified by the researchers.

### Outcome measures

#### Primary outcome

The primary outcome is CVAS change from baseline to the end of the follow-up phase (week 36). CVAS is used to assess the participants’ perceptions of the severity of their cough. Participants will be asked to draw a vertical line intersecting a 10-cm horizontal line to indicate the severity of their cough: zero is no cough while 10 cm is the worst cough imaginable [30].

#### Secondary outcomes

##### Quality of life

The total score in the Chinese version of the Leicester Cough Questionnaire [31] will be used to assess the quality of life of the participants (at weeks 1, 4, 8, 12, 24, and 36).

##### Lung function

Lung function will be determined mainly by measuring the forced expiratory volume in the first second (FEV1), the ratio of forced vital capacity occupied by forced expiratory volume in the first second (FEV1/FVC), and cumulative dose of bronchial activator to reduce FEV1 by 20% (PD20-FEV1) (at weeks 1, 12, 24, and 36).

##### Blood tests

The blood tests will include peripheral blood eosinophil count, eosinophil cationic protein, IgE, IFN-γ, and IL-4 (at weeks 1, 12, 24, and 36).

##### Other secondary outcomes

The rate of symptom relapse will be assessed at week 36. Overall health resource utilization will be recorded, including doctor visits, hospitalizations, and medication usage.

### Safety assessment

Blood and urine samples, liver function, renal function, electrocardiograms, and chest X-rays will be examined at the end of the run-in period and at weeks 4 and 12. Researchers will pay attention to abnormal changes in the results.

An AE is any adverse medical event that occurs during the trial, regardless of whether it is related to the drugs of the trial. Patients will be required to report all AEs at each visit. The effects, degree, time of occurrence, duration, treatment process, and follow-up of AEs will be recorded on case report forms. All AEs will be reviewed to assess the causal effects of the interventions, according to the standards set by the Uppsala Monitoring Centre of the World Health Organization [32].

### Termination and withdrawal

The trial will be terminated for any participant who develops one or more of the following conditions during the trial: (1) CVA develops into typical asthma, (2) intolerable side effects, or (3) serious acute or chronic organic disease. Any participant can withdraw from the trial for any reason at any time without prejudicing current or future treatments. The investigators will try to contact any withdrawn or terminated participants to complete the final assessment. Reasons for withdrawal or termination and the last medication time will be recorded. All withdrawn and terminated cases will be reported and analyzed.

### Compliance

The compliance evaluation will be conducted using the pill counting method. Each participant will be asked to keep a daily medication diary. Investigators will telephone the participants every 2 weeks to check their compliance. At the subsequent visit, the participants will show the investigator their diaries and report their compliance with the doctor’s advice. Ongoing support, such as free registration and treatment advice, will be provided to the participants in the follow-up phase.

### Sample size estimation

The sample size calculation for the primary outcome needs to be statistically and clinically significant. Based on previous literature [7] and our observational study, a sample size of 24 participants in each group can achieve 90% power to dismiss from a two-sided type I error of 0.05 to detect a superiority margin difference of 2.5 in this trial with two-tailed *P* values < 0.05 indicating significance. The total sample size was adjusted to 30 participants in each group to allow for 20% loss to follow-up and non-compliance.

### Data management and quality control

The investigators in the research team were required to attend a training workshop before recruitment. Each one received a copy of the study protocol and they were asked to adhere to the protocol during the study period. The investigators responsible for collecting data received training on how to perform CVAS and quality of life assessments. The lung function and blood tests will be done by medical professionals.

Data will be collected and recorded on case report forms. All data will be entered into a predesigned password-protected database by personnel blinded to group allocation. Data entry will be performed continually throughout the trial using the double-entry method, with any corrections or changes of data written in the case report forms documented and dated. Data quality will be checked regularly by a qualified specially assigned person. The Guangdong International Clinical Research Center of Chinese Medicine (Guangzhou, China), which is independent of the sponsor and does not have any competing interests, will be responsible for monitoring the data. The Department of Science Research of GPHCM, which is independent of the sponsor and investigators, will perform data audits in the middle of the trial.

### Statistical analysis

The data analysis will be carried out by an independent statistician using PASW Statistic 18.0 (IBM SPSS Inc., Armonk, New York, USA). Continuous data will be presented as means and standard deviations, or 95% confidence intervals. Dichotomous data, such as relapse of symptoms, will be presented as risk ratios and 95% confidence intervals. Two-tailed *P* values < 0.05 are considered to be statistically significant.

Baseline characteristics will be described using standard statistical analysis methods. The categorical variables will be analyzed via chi-squared tests or Fisher’s exact analyses, while continuous variables will be analyzed using *t*-tests. The main analyses include an intention-to-treat analysis and a per-protocol subject analysis for the primary outcomes. The last observation carried forward method will be used in the intention-to-treat analysis for missing data imputation. CVAS will be calculated as monthly averages, which will be compared with the average for the 2-week run-in period. Quality of life, lung function, and blood tests will be compared with the measurements from the end of the run-in period. Differences in changes from baseline to each time point between the two groups will be compared using analyses of variance. Paired *t*-tests will be used to compare data from before and after treatment in the same group. In the analysis of factors affecting the outcome changes, mixed-effects linear or logistic regression models will be performed, adjusting for baseline characteristics and other variables. Descriptive analyses and chi-squared tests or Fisher’s exact tests will be used for the safety data analysis. Any causality between AEs and the interventions will be taken into account.

## Discussion

To our knowledge, this is the first clinical study to investigate the long-term efficacy of CHM plus ICS/LABA for CVA. Compared to previous studies, we are focusing on CVAS, lung function, and airway inflammation, not only for the treatment phase but also during the 6-month follow-up phase.

In clinical practice, compliance with asthma medications is poor, especially for the inhaled glucocorticoids used for maintenance therapy. It is reported that only 52.4% of patients with CVA regularly inhaled ICS/LABA within the past 6 months [33]. This can lead to undertreatment of any underlying inflammation and increased risk of exacerbation. If the CHM granule plus ICS/LABA treatment is found to be safe and effective in the long term for CVA patients, it will be a great help in resolving this problem. The results of this trial will provide high-quality evidence of the efficacy of CHM in the treatment of CVA.

### Trial status

Recruitment into this study started on 1 January 2017, following protocol version 001/20160603. The final participants are expected to complete the 6-month follow-up assessment in mid-2018.

## Additional file

Additional file 1: SPIRIT Checklist. (DOC 118 kb)

## Abbreviations

AE: Adverse event
CHM: Chinese herbal medicine
CVA: Cough variant asthma
CVAS: Cough visual analog scales
FEV1: Forced expiratory volume in 1 s
GPHCM: Guangdong Provincial Hospital of Chinese Medicine
ICS/LABA: Inhaled corticosteroid (glucocorticoid) and long-acting β2-agonist
PEF: Morning and evening peak expiratory flow
SFC: Salmeterol/fluticasone propionate combination
